# Transient Midventricular Ballooning Syndrome: An Atypical Case of Stress Cardiomyopathy

**DOI:** 10.7759/cureus.18875

**Published:** 2021-10-18

**Authors:** Krupa K Solanki, Rishika Bajaj, Gaby B Aoun

**Affiliations:** 1 Department of Pulmonary and Critical Care, East Tennessee State University Quillen College of Medicine, Johnson City, USA; 2 Department of Internal Medicine, Johnston Memorial Hospital, Abingdon, USA; 3 Department of Interventional Cardiology, Saint Elizabeth Medical Center, Youngstown, USA

**Keywords:** takotsubo cardiomyopathy, stress-induced cardiomyopathy, midventricular ballooning syndrome, ventriculography, transthoracic echocardiogram, interventional cardiology

## Abstract

Stress cardiomyopathy can cause significant morbidity in the functional life of patients. The most common finding is apical ballooning of the left ventricle on cardiac catheterization. Some cases present with atypical imaging findings. This report presents a case of atypical stress cardiomyopathy with midventricular hypokinesis.

## Introduction

It is estimated that stress-induced cardiomyopathy is diagnosed in 1%-2% of cases of suspected acute coronary syndrome [1], and serious in-hospital complications occur for about one-fifth of afflicted patients [2]. An expert consensus on stress cardiomyopathy has recently established recommendations for treating this disease that has traditionally not had guidelines. This manuscript presents an atypical case of stress cardiomyopathy and attempts to illustrate the differences in its characteristics for clinical cardiologists who may see similar cases.

## Case presentation

A 63-year-old Caucasian female smoker with a notable past medical history of hypertension, gastroesophageal reflux disease, and depression was admitted to the hospital due to midsternal chest discomfort that radiated to her shoulders and neck. Her discomfort started approximately two and a half hours before she arrived at the emergency department. It was rated a 6/10 in intensity and was associated with nausea, sweating, and dizziness. Cardiovascular examination revealed a regular rhythm without murmurs or gallops. She had equal pulses in all extremities. The remainder of her physical examination was normal.

**Figure 1:**
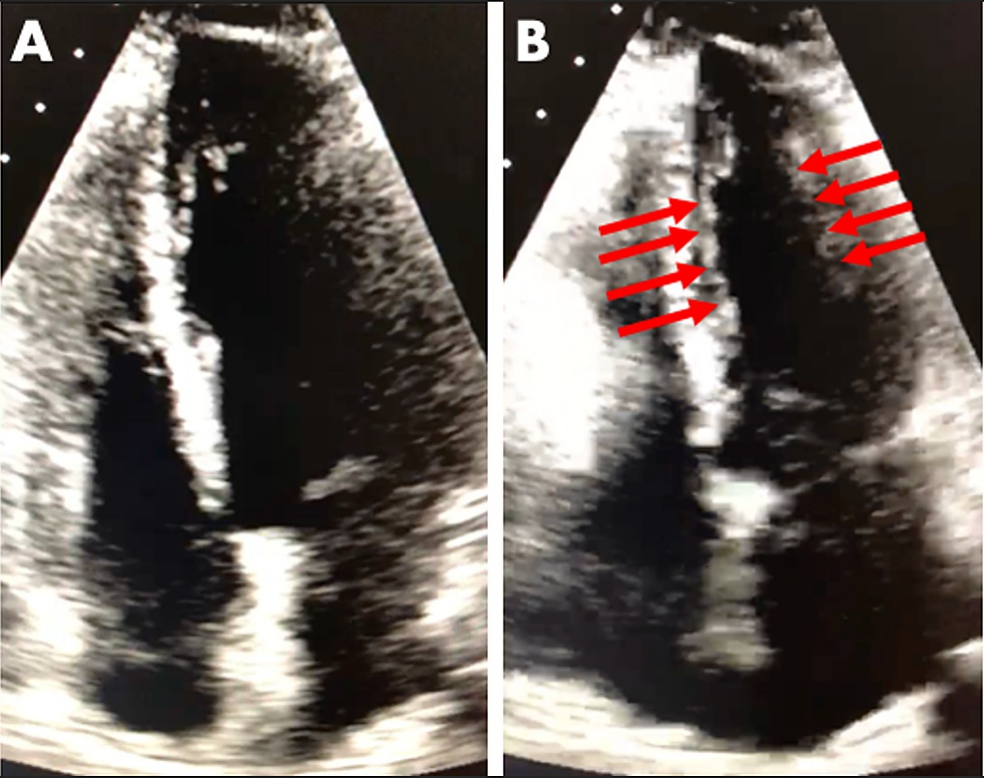
Transthoracic echocardiogram of the patient demonstrating midventricular ballooning syndrome. (A) Left ventricular diastole. (B) Severe hypokinesis of the midleft ventricular wall (red arrows) during systole.

The initial workup at the emergency department revealed severe hypertension (195/96 mm Hg). Two subsequent echocardiograms (ECGs) showed sinus bradycardia at 59 beats/minute, poor R-wave progression, and T-wave inversions in leads V1-V3. There was no QTc prolongation. Other laboratory abnormalities included an elevated troponin trend of 1.04, 1.54, and 1.11 ng/mL drawn six hours apart. She had an elevated cholesterol level of 231 mg/dL, an LDL level of 126 mg/dL, an HDL level of 79 mg/dL, and a triglyceride level of 150 mg/dL. Transthoracic echocardiogram demonstrated hypokinesis of the midleft ventricular wall during systole (Figure 1).

**Figure 2:**
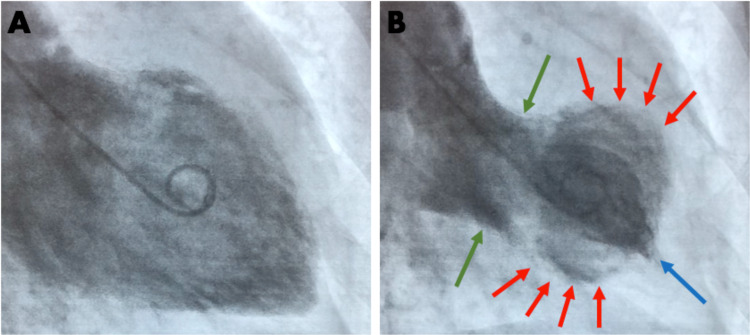
Left ventriculogram of the patient demonstrating midventricular ballooning syndrome. (A) Left ventricular diastole. (B) Hypokinesis of the midsegment of the left ventricle (red arrows) with full motion of the apical (blue arrow) and basal (green arrows) segments during systole.

The patient received 2 mg of morphine and 0.4 mg of sublingual nitroglycerin on arrival. Subsequent cardiac catheterization revealed 20% ostial stenosis in the left anterior descending artery and 20%-30% ostial stenosis in the ramus. There was no angiographically significant stenosis. However, a left ventriculogram revealed moderate to severe hypokinesis of the left midventricle that spared the apex and base (Figure 2). Her left ventricular ejection fraction was estimated to be 45%-50%. No mitral regurgitation was noted. She was discharged on aspirin and high-dose statin therapy.

## Discussion

Midventricular ballooning syndrome is an atypical presentation of stress cardiomyopathy in which there are transient wall motion abnormalities in the midsegment of the left ventricle rather than the classical apical presentation [3]. Similar to the typical stress cardiomyopathy (commonly referred to as “Takotsubo syndrome”), it is thought to be caused by increased sympathetic stimulation. It is usually emotionally triggered in females and physically triggered in males [4]. The exact mechanism of this myocardial stunning is still not well understood, but it is hypothesized to be the result of a toxic effect of catecholamines on cardiac myocytes [3]. The disease disproportionally affects women (~90%), the large majority of which are postmenopausal [4].

Atypical presentations of stress cardiomyopathy include instances with midventricular, basal, and focal wall motion abnormalities [5]; they are actually quite common and can represent up to 40% of cases [6]. Comparatively, these patients can present with lower brain natriuretic peptide levels and greater ejection fractions [7]. Atypical stress cardiomyopathies have also been shown to present in younger patients with less prevalence of hypertension [8], ST-segment elevation, and neurologic disease, but studies have shown no prognostic differences between atypical and typical variants [7,8]. Management is aimed at removing the offending stressor. A follow-up echocardiogram will usually show resolution of wall motion defects. However, serious complications, such as pulmonary edema, ventricular thrombi, cardiac rupture, cardiogenic shock, cardiac arrest, and death, can occur in up to 20% of patients and predominantly affect males [4].

There are no established guidelines for treating stress-induced cardiomyopathy, but it is recommended that patients with symptoms receive a full ischemic workup, including cardiac catheterization if necessary [2]. After diagnosis, many patients are usually treated with heart failure medications and antithrombotic therapy for up to 12 months [9]. Unlike beta-blockers, angiotensin-converting enzyme inhibitors have shown increased survival benefits [2,4]. Aspirin and statins may be given to patients with atherosclerosis on cardiac imaging. Psychiatric medications should also be used to treat comorbid depression and anxiety [2].

## Conclusions

Unlike typical stress cardiomyopathy, atypical stress cardiomyopathy spares the apex and has been shown to occur in younger patients with less incidence of hypertension, neurologic disease, and ST-segment elevation. There are no significant prognostic differences between these two variants of disease. Prognosis is usually favorable with eventual full resolution of wall motion defects demonstrated on serial imaging. Although there are no established treatment guidelines, heart failure medications and antithrombotic therapy are typically used. Angiotensin-converting enzyme inhibitors have been found to increase survival benefits. Careful consideration of the individual patient is necessary to tailor appropriate therapy.
